# Attenuation of *Zucchini mosaic virus* disease in cucumber plants by mycorrhizal symbiosis

**DOI:** 10.1007/s00299-023-03138-y

**Published:** 2024-02-05

**Authors:** Rabab A. Metwally, Mohamed A. Taha, Nada M. Abd El-Moaty, Reda E. Abdelhameed

**Affiliations:** 1 Botany and Microbiology Department, Faculty of Science, Zagazig University, Zagazig, 44519 Egypt; 2 Microbiology Department, Soil, Water and Environment Research Institute (SWERI), Agricultural Research Center, Giza, Egypt

**Keywords:** Mycorrhizal symbiosis, Pathogenesis-related genes, Viral infection, Cucumber, Disease severity, Proline

## Abstract

**Key message:**

Arbuscular mycorrhizal fungi generated systemic acquired resistance in cucumber to *Zucchini yellow mosaic virus*, indicating their prospective application in the soil as a sustainable, environmentally friendly approach to inhibit the spread of pathogens.

**Abstract:**

The wide spread of plant pathogens affects the whole world, causing several plant diseases and threatening national food security as it disrupts the quantity and quality of economically important crops. Recently, environmentally acceptable mitigating practices have been required for sustainable agriculture, restricting the use of chemical fertilizers in agricultural areas. Herein, the biological control of *Zucchini yellow mosaic virus* (ZYMV) in cucumber (*Cucumis sativus* L.) plants using arbuscular mycorrhizal (AM) fungi was investigated. Compared to control plants, ZYMV-infected plants displayed high disease incidence (DI) and severity (DS) with various symptoms, including severe yellow mosaic, mottling and green blisters of leaves. However, AM fungal inoculation exhibited 50% inhibition for these symptoms and limited DS to 26% as compared to non-colonized ones. The detection of ZYMV by the Enzyme-Linked Immunosorbent Assay technique exhibited a significant reduction in AM-inoculated plants (5.23-fold) compared with non-colonized ones. Besides, mycorrhizal root colonization (F%) was slightly reduced by ZYMV infection. ZYMV infection decreased all growth parameters and pigment fractions and increased the malondialdehyde (MDA) content, however, these parameters were significantly enhanced and the MDA content was decreased by AM fungal colonization. Also, the protein, proline and antioxidant enzymes (POX and CAT) were increased with ZYMV infection with more enhancements due to AM root colonization. Remarkably, defence pathogenesis-related (PR) genes such as PR-a, PR-b, and PR-10 were quickly expressed in response to AM treatment. Our findings demonstrated the beneficial function of AM fungi in triggering the plant defence against ZYMV as they caused systemic acquired resistance in cucumber plants and supported their potential use in the soil as an environment-friendly method of hindering the spread of pathogenic microorganisms sustainably.

**Supplementary Information:**

The online version contains supplementary material available at 10.1007/s00299-023-03138-y.

## Introduction

In natural ecosystems, plants simultaneously interact with a broad panel of microorganisms, both pathogens and symbionts, giving rise to a complex interaction that affects agricultural crop production (Rodriguez et al. 2019; Harman et al. 2021). From these pathogens, viruses represent a major threat to sustainable agriculture and global food security, resulting in huge losses in crop production and quality (Mumford et al. 2016; Tatineni and Hein 2023). Most viral diseases are characterized by systemic damage, in which the virus spreads from the primary site of inoculation to other parts of the plant until it dies off once the plant is infected. It is passed to the offspring by vegetative propagation (Anikina et al. 2023). Symptoms of viral infections typically include developmental abnormalities, necrosis, and chlorosis (Wang et al. 2022). Viral infection has been linked to a general decline in plant function, including suppression of photosynthesis (Rahoutei et al. 2001) and a drop in biomass (van Mölken and Stuefer 2011). The most damaging one is the *Zucchini yellow mosaic virus* (ZYMV), which is a member of the genus *Potyvirus*, (*Potyviridae*), and exhibits significant yield loss and destructive severe symptoms. *Cucurbitaceae* family crops such as cucumber, squash, pumpkin, and watermelon are the virus targets showing yellowing, blisters, severe mosaic, vein clearing, and leaf deformation (Radwan et al. 2007; Bubici et al. 2020). ZYMV is a single-stranded RNA genome surrounded by flexible rod particles in shape. Its RNA consists of about ~ 9.6 Kb (Balint et al. 1990), which has a poly (A) tail and a 5/viral protein genome linked (VPG) (Dougherty and Semler 1993).

Curing and controlling plants once they have been infected by a virus is not achievable, in contrast to bacteria or fungi that can be treated with antibacterial or antifungal agents. Therefore, managing disease focuses on keeping viruses out of plants or making plants resistant to viral infection. To do this, several tactics that are tailored to each virus, host and environment must be created (Rubio et al. 2020; Anikina et al. 2023). Agrochemicals are commonly used to suppress plant virus infection, but these methods are unacceptably expensive and may have negative environmental implications. Other effective tactics for limiting viral diseases include the development of virally tolerant or resistant crops (Nicaise 2014), introducing resistance genes from wild accessions, and using transgenic plants that express viral components that can disrupt viral infection mechanisms at the RNA or protein level (Faoro and Gozzo 2015). Unfortunately, none of these tactics can be used right away to manage viral infections. To control the viral infection and limit the use of chemical fertilizers in agricultural areas, it is important to establish an environmentally acceptable strategy. Thus, the potential for improving plant immunity to viruses by utilizing advantageous microorganisms, such as arbuscular mycorrhizal (AM) fungi, merits careful consideration and has been suggested as a practical and sustainable approach to suppress plant viruses (Hao et al. 2019).

AM fungi are a class of obligate biotrophs that may colonize the roots of many plant species, including food crops, and assist plants in overcoming abiotic and biotic challenges on agricultural soils (Spatafora et al. 2016; Abdelhameed and Metwally 2018, 2019; Zhao et al. 2022; Wu et al. 2023). Even while AM fungi are morphologically restricted to the roots, the physiological and metabolic changes they elicit in the root also affect the physiology of the plant as a whole (Abdelhameed and Metwally 2018, 2019; Metwally 2020). Due to their reliance on lipids and carbohydrates supplied by plants, fungal symbionts serve as powerful carbon sinks in roots. As a result, the regulation of photosynthesis and leaf primary metabolism maintains the carbon balance in plants (Kaschuk et al. 2009; Metwally et al. 2021; Wu et al. 2023). The fungus uses arbuscules, which are highly branching internal fungal structures, to facilitate the passage of mineral nutrients, such as phosphate, to the root cells (Bonfante 2001; Metwally and Abdelhameed 2024). In addition to providing nutrition, physiological alterations that cause metabolic changes in the root by way of AM fungi can also improve the host's resistance to environmental stresses like drought (Salam et al. 2017; Begum et al. 2021), salinity (Metwally and Abdelhameed 2018), heavy metals (Abdelhameed and Metwally 2019), and biotic stress like pathogen attack on roots and aerial organs (Harrier and Watson 2004; Sarkar and Sadhukhan 2023).

ZYMV, like many plant viruses, can be transmitted through seeds, posing a significant challenge in viral disease management. The seed transmission rate of ZYMV is a critical parameter influencing the spread and persistence of the virus within the plant population (Desbiez and Lecoq 1997; Simmons et al. 2013). The potential mechanisms through which AM fungi could reduce seed transmission of ZYMV may include: (1) Induction of systemic acquired resistance (SAR) via the stimulation of pathogenesis-related (PR) proteins in plants (Romera et al. 2019), and activating defense mechanisms that can extend to the reproductive structures, reducing the likelihood of ZYMV transmission to seeds. (2) Competition for resources, as AM fungi may indirectly compete with the virus for essential resources within the plant. The potential role of AM fungi in reducing the seed transmission of ZYMV opens up intriguing avenues for further research and practical applications in agricultural settings. Investigating the specific mechanisms involved in the interaction between AM fungi and ZYMV, as well as their impact on seed transmission rates, could provide valuable insights for the development of sustainable and integrated strategies to manage viral diseases in crops (Simmons et al. 2013; Hu et al. 2018).

AM fungi also provide a physiological state that enables plants to react more quickly and forcefully to pathogen attacks. According to Mendoza-Soto et al. (2022), tomato plants with AM colonization are less vulnerable to the *Tomato mosaic virus* (ToMV). Maffei et al.’s study (2014) showed a clear protective role of AM fungi against viral infections in roots and shoots and in disease symptoms, where during the early stages, decreased or no difference in the severity of symptoms was detected in AM plants compared to non-AM ones. Moreover, Hao et al. (2018) displayed that AM colonization greatly reduced gall formation on roots and nematode vector *Xiphinema index* multiplication in soil, while also shielding grapevines from the *Grapevine fanleaf virus* (GFLV). On the other hand, while investigating the interactions between *Funneliformis mosseae* and *Tomato spotted wilt virus* (TSWV) in tomato, Miozzi et al. (2011) found no differences in virus accumulation or symptom severity between AM and non-AM plants. These variable results concerning viral infections and AM plants depend on how the plant, AM fungus, and virus interact with one another.

Nevertheless, reports on the biocontrol ability of the AM fungus against viral plant diseases are scarce. The induction of cucumber plant defenses against ZYMV by using biocontrol agents is essential for developing new strategies against these pathogens. Cucumber (*Cucumis sativus* L.) is an economically important plant and one of the top 10 vegetable crops worldwide (Kim et al. 2019). So, our study's goals were to determine how AM fungal symbiosis affected how severe ZYMV infection was in cucumber plants, as well as their effects on morphological and biochemical parameters. Furthermore, it elucidated the relationship between viral resistance and PR protein expression levels in response to AM fungal colonization.

## Materials and methods

### Biological materials

#### AM fungal inoculum

The AM fungal spores were initially isolated from the soil of El-Sharkia Governorate, Egypt, via wet sieving and decanting techniques then mounted using alcohol polyvinyl lactoglycerol (Gerdemann and Nicolson 1963). The spore combination was multiplied in pots with sterilized sandy clay soil and mounted in the greenhouse with Sudan grass (*Sorghum sudanenses* Pers.) roots as an acceptable trap plant for two cycles of 5 months. After multiplication, the AM fungal inoculum was obtained, and it included spores, soil, colonized root fragments (85%, colonization index), and hyphae. The used AM fungi were *Funneliformis mosseae*, *F. constrictum*, *Gigaspora margarita* and *Rhizophagus irregularis*.

#### Viral inoculum (Zucchini yellow mosaic virus)

**Fig. 1:**
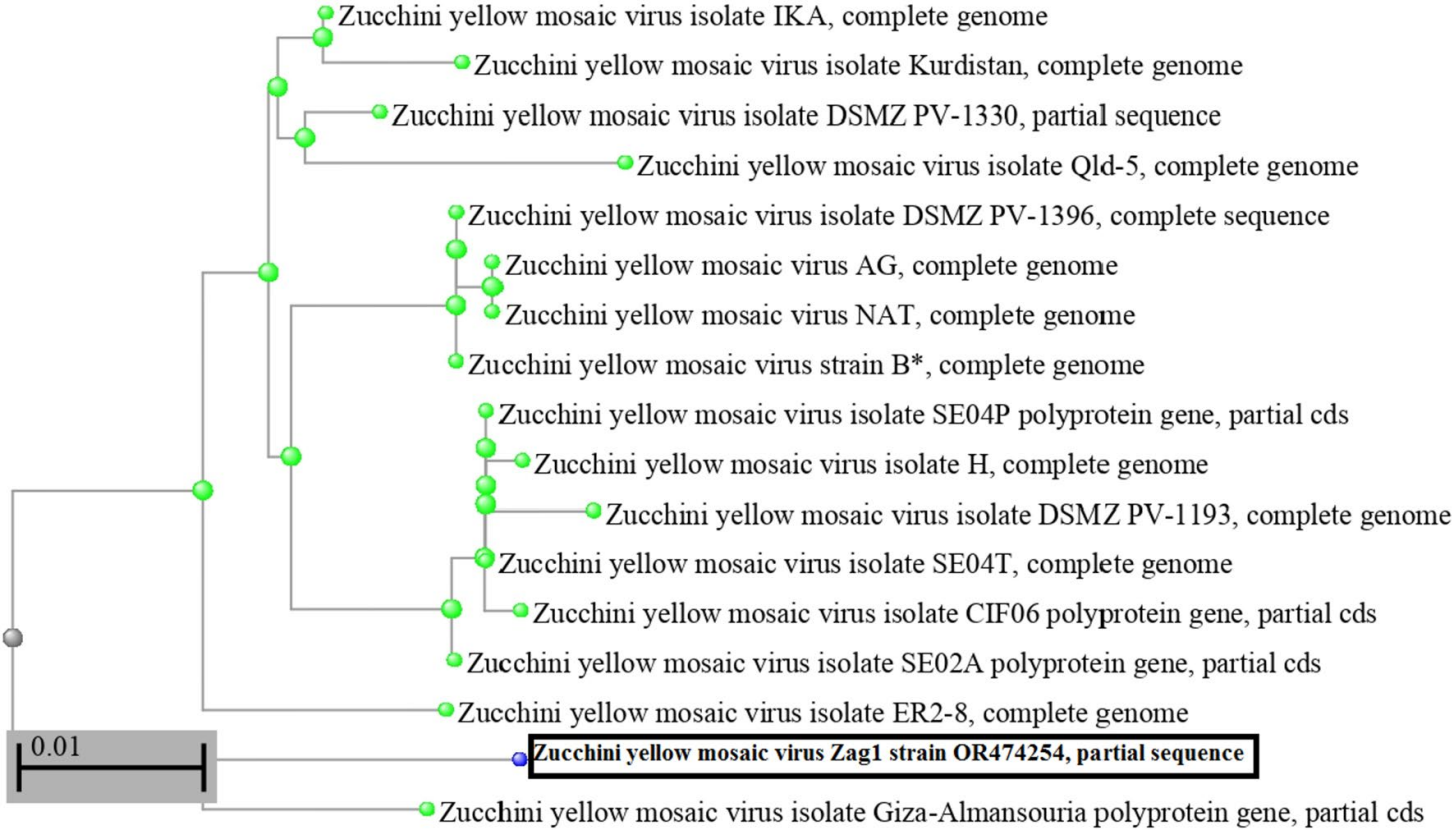
The phylogenetic tree of ZYMV-Zag1 strain deposited in the GenBank under accession No. OR474254

The ZYMV-Zag1 strain utilized in this investigation was identified molecularly and deposited in GenBank with the accession number OR474254, and the phylogenetic tree was constructed based on partial nucleotide sequences using neighbor-joining method (Fig. 1). ZYMV was first obtained from the Virology Laboratory, Faculty of Agriculture, Ain Shams University, Hadayek Shobra, Cairo, Egypt, where it was mechanically maintained on squash (*Cucurbita pepo* L.) leaves under an insect-proof greenhouse. The virus was mechanically inoculated onto *Chenopodium amaranticolor* leaves for the development of chlorotic local lesions. A single local lesion formed 6 days post-virus inoculation (dpi) was posteriorly used as a ZYMV source. The ZYMV inoculum was prepared by grinding infected *Chenopodium amaranticolor* leaves in 0.01 M sodium phosphate buffer, pH 7.0, 1:2 (W/V), and then filtered through two layers of cheese cloth.

### Plant material and potting medium

Cucumber (*C. sativus* L.; HAYEL, Hybrid cucumber) was used as the test plant, and its seeds were provided by the Agriculture Research Center, Giza, Egypt. Firstly, seeds were disinfected with 2% (w/v) NaOCl for 3 min, rinsed twice with sterilized distilled water, and dried up on sterilized filter paper. Cucumber seeds were propagated into sterilized plastic pots (three per pot; 25 cm in diameter), filled with 2 kg of sterilized soil that sterilized once every three sequential days in autoclave for 1 h at 121 ℃. Two groups of pots were applied during the sowing of seeds: the 1st group was AM- inoculated, and the 2nd one was non-AM inoculated. AM fungal inoculation was achieved by placing 20 g of AM fungal inoculum for AM-inoculated plants. Non-AM plants received 20 g of sterilized soil. The soil was of clay texture with a pH of 8.24, electric conductivity of 3.45 S/m, organic matter of 1.24%, total phosphorus of 0.69%, and a mineral content of K = 0.37, Mg = 6.34, and Ca = 8.47 mg/kg soil.

### Greenhouse study and tissue collection

The experiment was conducted in a controlled greenhouse environment [25/16 ℃ (day/night) with 70% relative humidity]. On the 20th day after planting, the leaves of cucumber plants were dusted with carborundum (600 mesh) and mechanically inoculated with the ZYMV. Using a fully randomized experimental design, four treatments were included; each one had five replicates. The 1st treatment was uninfected control cucumber plants without AM fungi that were sprayed with sterile distilled water (HC: healthy control). The 2nd one was uninfected cucumber plants inoculated with AM fungi (AM). The 3rd one was ZYMV-infected plants without AM fungi (ZYMV). The 4th one was ZYMV-infected cucumber plants with AM (AM + ZYMV). At 20 dpi, with daily observation of the development of viral symptoms, cucumber plants were collected for further analysis.

### Measurements

#### Disease incidence (DI) and disease severity (DS) assessment

At 20 dpi, the percentage of infected cucumber plants (DI) and the severity (DS) of ZYMV symptoms were assessed using the following scale: 0 = no symptoms; 1 = mild mosaic; 2 = mottling; 3 = mild mosaic, mottling, and blisters; 4 = severe mosaic, green blisters, and vein banding; and 5 = severe mosaic, green blisters, vein banding, and filiform. The disease severity was calculated according to the formula of Yang et al. (1996).

$$\% {\text{Disease incidence}} \,\,\left( {{\text{DI}}} \right) = \frac{{\left( { {\text{Number of infected plants in the treatment}} } \right)}}{{\left( { {\text{Total number of plants in the same treatment}}} \right)}} \times 100$$

$${\text{Disease severity }}\left( {{\text{DS}}} \right) \, = \,\frac{{\sum {\left( {{\text{disease grade }} \times {\text{ number of plants in each grade}}} \right)} }}{{{\text{Total}}\,{\text{number}}\,{\text{of}}\,{\text{plants}} \times \,{\text{highest}}\,{\text{disease}}\,{\text{grade}}}} \times 100$$

#### Evaluation of growth parameters

At 20 dpi, cucumber plants were divided into shoots and roots; roots were washed by water to remove soil particles. Their lengths and fresh weights (fw) were measured, and their leaves were numbered. After oven drying at 70 ℃ for 48 h, the dry weights of shoots and roots were also measured.

#### Photosynthetic pigments determination

According to Metzner et al. (1965), cucumber leaf samples (0.25 g fw/sample) were extracted in 85% acetone and then centrifuged at 8000 rpm for 10 min. The absorbance of the supernatants was recorded at 452.5, 644, and 663 nm using a UV–vis spectrophotometer using 85% acetone as the blank. The concentrations of pigments (mg/g fw) were calculated based on Lichtenthaler and Wellburn’s (1985) equations.

#### Mycorrhizal evaluation

Randomly selected roots from AM-inoculated plants (AM and AM + ZYMV) were first washed with water, cut into small pieces, and immediately cleared with 10% KOH. They were stained with 0.5% (w/v) trypan blue overnight, as described by Phillips and Hayman (1970) and observed under a light microscope. The percentage of root colonization was determined by observing the hyphae, arbuscules, and vesicles in these root segments (Trouvelot et al. 1986).

### ZYMV detection

#### DAS-ELISA (Double Antibody Sandwich-Enzyme Linked Immunosorbent Assay)

ZYMV concentration in the infected and healthy cucumber leaves was done according to Clark and Adams (1977) at 20 dpi by the DAS-ELISA technique. The absorbance readings were calculated at 405 nm using a microplate reader (Anthos 2010, Biochrom Ltd., Cambridge, UK). If the absorbance readings were greater than double the mean absorbance value of the HC, a positive ZYMV infection was assumed.

#### Transmission electron microscope (TEM)

Healthy and infected (developing severe symptoms of ZYMV) cucumber leaves were ground separately in 0.01 M sodium phosphate buffer (PB), pH 7.0. Small drops of each sap were dipped on carbon-coated grids for 1 min. Virus particles that existed on grids were stained using 2% sodium phosphotungstate on a glass slide, air dried for 1 min and then examined using TEM (JEOLJEM-100S) in the Electron Microscope Unit of the Egyptian Organization for Biological Products and Vaccines (VACSERA) (Agouza, Giza, Egypt), according to Wang and Li (2017).

### Determination of malondialdehyde (MDA) content

After extracting the MDA from cucumber leaves (0.25 g fw) using 10 mL of 0.1% trichloroacetic acid (TCA), the extracts were centrifuged for 15 min (Dhindsa et al. 1981). Two-milliliter aliquots of the supernatant were mixed with 2 mL of 20% TCA containing 0.5% thiobarbituric acid. The mixture was heated at 100 ℃ for 30 min, quickly cooled, and then centrifuged for clarification. At 532 and 600 nm, the absorbance was measured.

### Determination of proline content

Using 3% (w/v) aqueous sulphosalicylic acid, proline in cucumber leaves (0.25 g fw) was extracted (Bates et al. 1973), then 2 mL of supernatant was mixed with 2 mL of glacial acetic acid and 2 mL of acid ninhydrin reagent and incubated at 100 ℃ in a water bath for 1 h. The mixture was placed in an ice bath to stop the reaction, and toluene extraction was then performed. At 520 nm, the absorbance was measured, and a proline standard curve was used to determine the proline concentration (µmols/g fw).

### Protein and antioxidant enzymes assay

Cucumber leaves (1 g fw) were homogenized in 0.05 M cold PB (pH 7.0) containing 1 mM EDTA (Ethylene Diamine Tetra Acetic Acid). The supernatant was employed as a source of proteins and enzymes after centrifugation. The total soluble protein content was determined using the Folin-Ciocalteu reagent at 700 nm (Lowry et al. 1951) and its concentration (mg/g fw) was expressed using bovine serum albumen as the reference. Peroxidase (POX) and catalase (CAT) activities in cucumber leaves were assayed separately in 250µL of enzyme extract at wavelengths of 470 and 240 nm, according to Chance and Maehly (1955) and Aebi (1984), respectively.

### PR-genes analysis in cucumber by real time-polymerase chain reaction (RT-PCR)

Four treatments of cucumber plants [1st: control healthy (HC), 2nd: AM-inoculated (AM), 3rd: ZYMV-infected, and 4th: AM-inoculated and ZYMV-infected (AM + ZYMV)] were performed for the expression of selected defense-related genes. Total RNA was extracted from cucumber leaves (600 mg fw/ sample) at 20 dpi, ground to a fine powder in liquid N_2_, and stored at − 70 °C until used. An investigation of gene expression was performed at the Animal Health Research Institute in Giza, Egypt.

Following the method described by Suzuki et al. (2004), total RNA was extracted using the GeneJET Plant RNA Purification Mini Kit (Thermo Scientific, Germany). The sample was homogenized with the following extraction buffer: 100 mM Tris–HCl (pH 9.5), 10 mM EDTA (pH 8.0), 2% lithium dodecyl sulphate, 0.6 M NaCl, 0.4 M trisodium citrate, and 5% 2-mercaptoethanol were then centrifuged at 14,000 rpm for 5 min, after which the aqueous phase was re-extracted with a mixture of chloroform:isoamyl alcohol (24:1). Chloroform, sodium acetate (pH 4.0), guanidium thiocyanate, and water-saturated phenol were used to extract the supernatant. Following the manufacturer's instructions, the precipitated RNA was washed, air-dried, and treated with RNase-free water, DNase, and inactivation of the DNase. The RNA was reverse-transcribed using a cDNA synthesis kit (Fermentas, United States). The oligonucleotide primers used for detection are specified in Table 1 and were provided by BioResearch (Denmark). The transcript accumulation of each gene was normalized to PP2A as an internal reference, and the standard curve method was employed to quantify relative gene expression (Wu et al. 2014). The Stratagene Mx3005P software was used to calculate CT values and amplification curves. According to the “ΔΔCt” approach described by Yuan et al. (2006), the normalized ΔCT data were utilized to determine the relative gene expression fold change using a control calibrator (reference).

**Table 1:** Primers sequences, target genes and cycling conditions for SYBR green rt-PCR

Target gene	Primers sequences	Reverse transcription	Primary denaturation	Amplification (40 cycles)			Dissociation curve (1 cycle)			References
				Secondary denaturation	Annealing (optics on)	Extension	Secondary denaturation	Annealing	Final denaturation	
PRa	TGTGTTCCTGTTGCTGAATGTT	50 ℃ 30 min	94 ℃ 15 min	94 ℃ 15 s	60 ℃ 30 s	72 ℃ 30 s	94 ℃ 1 min	60 ℃ 1 min	94 ℃ 1 min	Wu et al. (2014)
	GGCGTTGGAATAATGAAGGTAG									
PRb	GGCCTCCAAGCAATTCTCCTCT									
	CCATTTAAAGGACATGCTGCTAC									
PR10	GAAGAAGAACACAATGAAGGCA									
	CAGTAGGATTGGCAAGAAGGTA									
PP2A (house keeping gene)	GACCCTGATGTTGATGTTCGCT									
	GAGGGATTTGAAGAGAGATTTC									

### Statistical analyses of the data

The reported experiment data are mean values ± standard error (SE) for each treatment. The programme SPSS 10.0 for Windows was used to conduct the statistical analysis, and one-way analysis of variance (ANOVA) was used to determine the main effects (viral infection and AM inoculation and their interactions). The comparisons among means were assessed using Duncan’s test calculated at *p* < 0.05.

## Results and discussion

### Phenotypic responses of mycorrhizal plants to ZYMV infection (DI and DS)

**Fig. 2:**
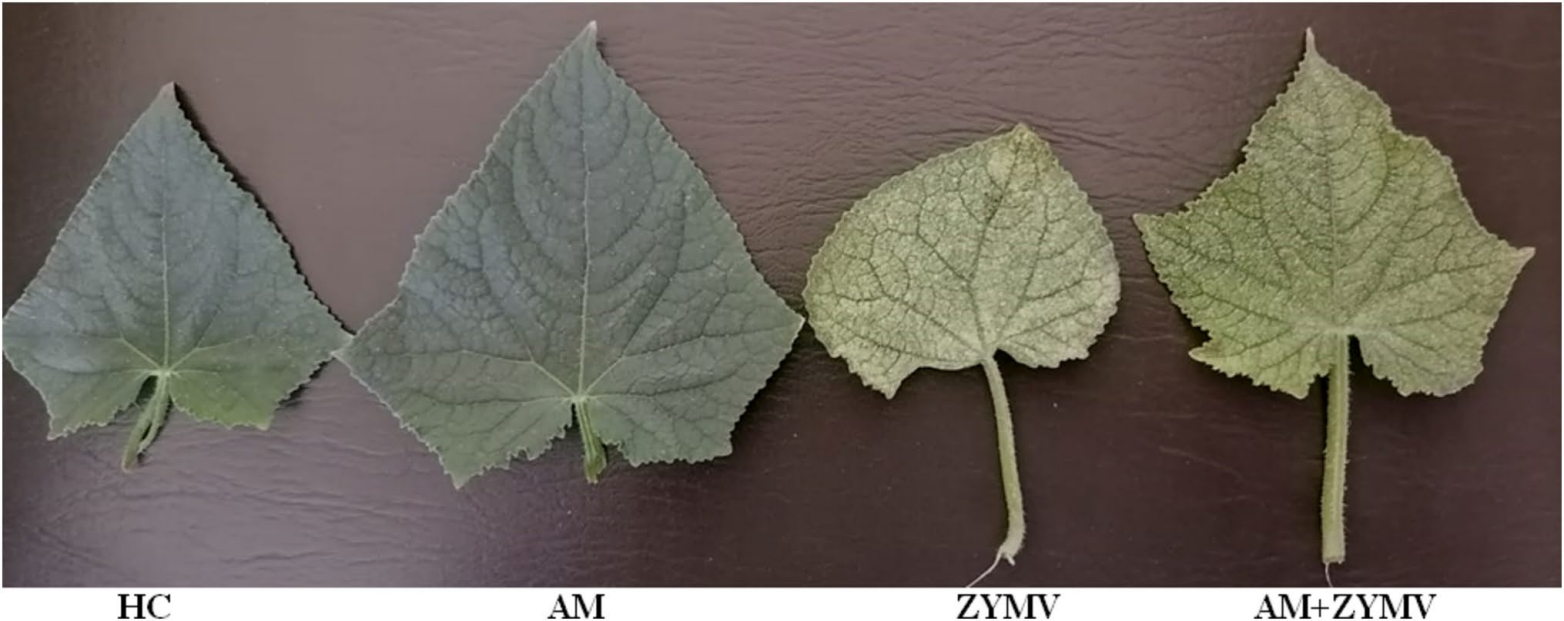
A photograph showing the disease symptoms on cucumber leaves infected with ZYMV in response to AM fungal colonization. *HC* Healthy control, *AM* plants colonized with AM fungi, *ZYMV* = ZYMV-infected plants and AM + ZYMV = AM and ZYMV-infected cucumber plants

Utilizing an eco-friendly and safe strategy such as AM fungi is a convincing way for fortifying plants to be protected against pathogens, particularly plant viral ones. Similar to other pathogens, viral infection causes many morphological, physiological, and biochemical alterations within the plant (Radwan et al. 2007). Consequently, disease symptoms on the cucumber leaves were manifested in Fig. 2. The inhibition rate, or DI, and the mean of DS percentages were assessed to detect the effect of AM inoculation on induction resistance against ZYMV in cucumber. The various disease symptoms of ZYMV appeared on cucumber leaves, either AM-inoculated or not, at 20 dpi, as shown in Fig. 2. ZYMV-infected cucumber plants and non-AM inoculated displayed a severe yellow mosaic, mottling, and green blisters of leaves as compared to AM-inoculated ones. The infected leaves also presented a fan-shaped appearance. AM plants infected by ZYMV constantly showed a significant alleviation of symptoms, with a mild mosaic and light mottling symptoms being more prevalent than in non-AM ones (Fig. 2). At 20 dpi, under ZYMV infections, there was a significant reduction (Table 2) in DI in AM-inoculated cucumber plants (50%) relative to non-AM ones (90%). Moreover, significant differences were observed in the DS values of ZYMV among AM-inoculated plants (26%) as compared with those non-inoculated (96%).

**Table 2:** Disease assessment and virus concentration (DAS-ELISA) of cucumber plants infected with ZYMV (20 dpi) in response of AM colonization

Treatments	Disease incidence (DI) (%)	Grade of DI	Disease severity (DS) (%)	Virus concentration
HC	0 ± 0.0c	–	0 ± c	0.086 ± 0.0029c
AM	0 ± 0.0c	–	0 ± c	0.044 ± 0019c
ZYMV	90 ± 2.31a	High	96 ± 2.53a	1.869 ± 0.055a
AM + ZYMV	50 ± 1.99b	Moderate	26 ± 0.687b	0.357 ± 0.009b

*HC* Healthy control, *AM* plants colonized with AM fungi, *ZYMV* ZYMV-infected plants and AM + ZYMV = AM and ZYMV-infected cucumber plants

^*^Values of each column followed by the same letter are not significantly different according to Duncan’s multiple range test (*p* ≤ 0.05), each value represents the mean of five replicates ± SE. a, b, c and d: symbols of significance letters

Our results highlight the biocontrol efficacy of mycorrhization, which serves as a tolerance inducer, protecting cucumber plants against ZYMV. This protection is evidenced by a decrease in DS and the alleviation of viral symptoms. As indicated in Table (2), AM inoculation achieves a 44.44% decrease in DI and is able to limit DS to 26% as compared to an infected one. Similarly, Khoshkhatti et al. (2020) found that *Rhizoglomus irregularis* inoculation of tomato plants significantly decreased the *Tomato bushy stunt virus* (TBSV) symptoms of young plant leaves as compared to non-inoculated ones at 20 dpi. Also, Miozzi et al. (2020) investigated the effect of *F. mosseae* colonization on tomato infection with cucumber mosaic virus (CMV) at 14 dpi. They demonstrated that infected plants had much more severe viral symptoms and had a threefold higher average DS than AM plants. In a previous study, AM colonization induced tomato-acquired resistance with decreasing DS of *Tomato yellow leaf curl Sardinia virus* (TYLCSV) symptoms, which gave an explanation of the fungal role of AM as a bio-protection agent against viral diseases (Maffei et al. 2014).

### Effects of ZYMV and mycorrhization on the growth parameters

To more accurately assess the impact of AM colonization on viral infection, the morphological parameters of cucumber plants were measured (Table 3 and supplementary Fig. 1). AM-inoculated plants exhibited enhanced growth relative to non-inoculated ones. ZYMV infection markedly attenuated this AM-induced growth enhancement, particularly in the roots. This was evident through a significant decline in the lengths and fresh and dry weights of cucumber shoots and roots when contrasted with their healthy counterparts. These reductions in growth of ZYMV-infected cucumber plants might be due to viral infection, which profoundly alters plant metabolism and reduces photosynthetic activity, which hinders the metabolism of sugars and other compounds (Anikina and Seitzhanova 2015).

**Table 3:** Effect of AM root colonization on the growth parameters of cucumber plants in response to ZYMV infection

Treatments	Shoot length (cm/plant)	Shoot fw (g/plant)	Root fw (g/plant)	Shoot dw (g/plant)	Root dw (g/plant)	Leaves number(No. /plant)
HC	36 ± 0.952b	15.3 ± 0.4048b	1.35 ± 0.0357b	1.0826 ± 0.0286b	0.0361 ± 0.00095b	7 ± 0.185b
AM	41 ± 1.085a	18.9 ± 0.5a	2.068 ± 0.054a	1.568 ± 0.0415a	0.0548 ± 0.00145a	8 ± 0.2116a
ZYMV	24.5 ± 0.648d	11.2 ± 0.296c	0.5999 ± 0.0158d	0.79 ± 0.021c	0.0215 ± 0.00056d	6 ± 0.1587c
AM + ZYMV	30 ± 0.794c	14.4 ± 0.38b	0.8899 ± 0.02c35	0.877 ± 0.023c	0.0299 ± 0.00079c	7 ± 0.185b

*HC* Healthy control, *AM* plants colonized with AM fungi, *ZYMV* ZYMV-infected plants and AM + ZYMV = AM and ZYMV-infected cucumber plants

^*^Values of each column followed by the same letter are not significantly different according to Duncan’s multiple range test (*p* ≤ 0.05), each value represents the mean of five replicates ± SE. a, b, c and d: symbols of significance letters

However, greenhouse data showed that AM root colonization greatly improved all growth metrics measured, recording the best results in comparison to the other treatments, where the length, fresh weight and dry weight of the shoot increased by 13.88, 23.52, and 44.83%, respectively, in comparison to a healthy, non-colonized one (Table 3). Most noticeably, AM fungal colonization of ZYMV-infected cucumber plants significantly reduced the negative effects of ZYMV on growth parameters compared to non-inoculated plants. This outcome was consistent with Maffei et al.’s (2014) finding that TYLCSV-infected tomato plants colonized with AM fungus showed a notable decrease in disease symptoms. In a similar respect, Thiem et al.’s (2014) investigation looked at the impact of *R. irregularis* colonization on potato plants infected with *Potato virus Y* (PVY), which showed milder symptoms in terms of shoot growth. Additionally, when compared to non-AM plants, tobacco and cucumber plants colonized by *R. irregularis* and infected with *Tobacco mosaic virus* (TMV) and *Cucumber green mottle mosaic virus* (CGMMV), respectively, showed enhanced growth, diminished disease symptoms, and lower viral titers (Stolyarchuk et al. 2009).

The elucidation for these findings is that AM fungal association enhances the uptake of water and nutrients by cucumber plants through the presence of extensive extra-radical hyphal networks in soils that are more thin than roots and can pass through smaller pores; they obtain macronutrients (such as orthophosphate) and micronutrients (such as Zn^2+^) from soil that is unobtainable to roots (Hao et al. 2019; Metwally and Abdelhameed 2019; Abdelhameed and Metwally 2022). Also, these fungi aid in the improvement of morphological and physiological mechanisms. On the other hand, it is understood that a strong plant is better able to fend off invading diseases than a weak one, and plant growth promotion by AM fungus plays a significant part in making up for disease damages.

### Effects of ZYMV and mycorrhization on the photosynthetic pigments

**Fig. 3:**
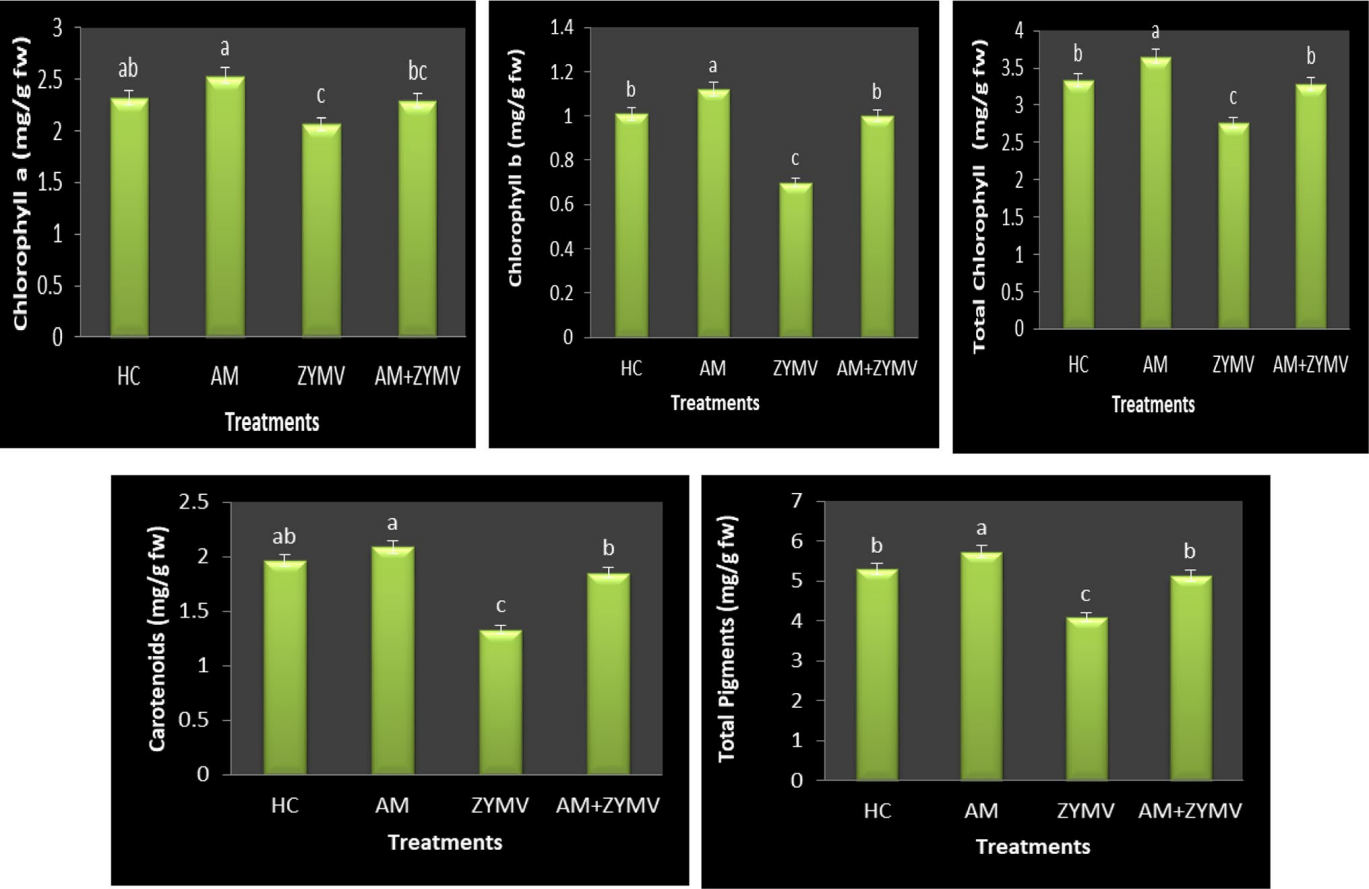
Effects of ZYMV infection and AM root colonization on the photosynthetic pigments contents (mg/g fw) of cucumber leaves. HC = Healthy control, *AM* plants colonized with AM fungi, *ZYMV* ZYMV-infected plants and AM + ZYMV = AM and ZYMV-infected cucumber plants. *Values of each column followed by the same letter are not significantly different according to Duncan’s multiple range test (*p* ≤ 0.05), each value represents the mean of three replicates ± SE. a, b, c and d: symbols of significance letters

Where chlorotic and necrotic symptoms manifest in nearly all virus-infected plants, there is a decrease in the net photosynthetic rate and consequently in the Chl content (Venkatesan et al. 2010). Figure 3 displays the means of the photosynthetic pigments of cucumber plant leaves in response to the various applied treatments. In response to ZYMV infection, leaves' Chl a, Chl b, carotenoids, and total pigments dramatically dropped to about 11.15, 30.62, 32.36, and 22.73% in comparison to the HC. Similar findings were made by Técsi et al. (1996) and Radwan et al. (2007), who noted the emergence of chlorosis and a decline in photosynthesis. Also, these pigments were shown to be declining in *Vigna mungo* plants that were infected with the *Mung bean yellow mosaic india virus* (MYMIV), according to Kundu et al. (2013). When mesta and cotton plants were infected with *Cotton leaf curl burewala virus* (CLCuV) and *Mesta yellow vein mosaic virus* (MeYVMV)), respectively, similar reductions in green pigments were seen in both plant species (Chatterjee and Ghosh 2008; Siddique et al. 2015). Suhail et al. (2020) also noted a reduction in Chl a, b, and carotenoids in MYMIV-infected plants of three mung bean cultivars. It is well recognized that plant viruses, which cause systemic infections, may be particularly known as inhibitors of chl production because they proliferate continually during plant growth and development (Sutic and Sinclair 1990). Chloroplasts are damaged and aggregated as a result of viral infection, which causes Chl to be destroyed or to stop synthesizing. The properties of the virus strain, the disease development phase's characteristics, the host plant's characteristics, and the environmental variables all play a role in how much photosynthetic suppression occurs (Akbar et al. 2021).

Most intriguingly, compared to colonized plants, the colonization of cucumber plants with AM fungus considerably increased all measured pigments, recording the highest levels. In ZYMV-infected plants, AM root colonization caused an increase of 10.96% Chl a, 42.85% Chl b, and 39.27% carotenoids compared to non-colonized ones (Fig. 3). According to Aseel et al.'s research (2019), compared to uncolonized plants, the photosynthetic pigments of ToMV-infected plants were greatly improved by AM colonization. AM fungal application increased the pigment content of cucumber plants, which may be attributed to increased stomatal conductance and carbon uptake or the increase in P and Mg^2+^ uptake by extra-radical mycorrhizal hyphae, which are crucial components required for photosynthesis (Metwally and Al-Amri 2019; Abdelhameed et al. 2021a).

### Effect of ZYMV on mycorrhizal colonization

**Fig. 4:**
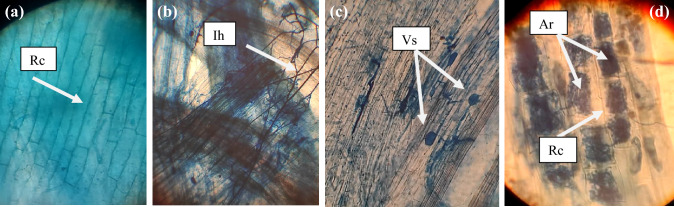
Cucumber root showing typical mycorrhizal colonization structures. Non-AM colonized cucumber root cells (**a**), and AM-colonized root (**b-d**). Where, *Rc* root cell, *Ih* intra-radical hyphae, *Vs* vesicles and *Ar* arbuscules

As viral infection can influence mycorrhization, the levels of AM colonization in cucumber roots of the applied treatments (AM and AM + ZYMV) are presented in Table 4. The efficiency of colonization was determined by counting the fungal structures in the colonized plant roots. Mycorrhizal symbiosis was well-established in all AM-inoculated plants and non-existent of it in non-inoculated plants (Fig. 4a). Light microscopy analysis showed the presence of both arbuscules and intra-radical mycelium in the root cortex of the colonized plants, which confirms successful colonization (Fig. 4b–d). The arbuscules act as primary structures that play a role in nutrient exchange and transport between the fungus and the plant. The evaluated colonization rates (colonization frequency (F%), intensity (M%), and arbuscules frequency (A%)) were found at high levels in the roots of AM-colonized, uninfected plants, registering 92.5, 49.2, and 23.4%, respectively.

**Table 4:** Mycorrhization levels of AM and ZYMV-infected AM plants were assessed according to the method described by Trouvelot et al. (1986)

Treatments	F%	M%	A %
HC	0 ± 0.0b	0 ± 0.0b	0 ± 0.0b
AM	92.5 ± 2.54a	49.2 ± 1.04a	23.4 ± 0.608a
ZYMV	0 ± 0.0b	0 ± 0.0b	0 ± 0.0b
AM + ZYMV	89.6 ± 2.33a	46.3 ± 1.01a	21.6 ± 0.502a

F%, frequency of root colonization; M%, intensity of cortical colonization and A%, arbuscules frequency in the root system

*HC* Healthy control, *AM* plants colonized with AM fungi, *ZYMV* ZYMV-infected plants and AM + ZYMV = AM and ZYMV-infected cucumber plants

^*^Values of each column followed by the same letter are not significantly different according to Duncan’s multiple range test (*p* ≤ 0.05), each value represents the mean of five replicates ± SE. a, b, c and d: symbols of significance letters

An additional result from our study **(**Table 4) was that ZYMV infection shows no clear effect or no significant difference in AM root colonization in ZYMV-infected plants as compared to healthy ones. In this regard, Maffei et al. (2014) found that *Tomato yellow leaf curl virus* (TYLCV) infection had no effect on the incidence of mycorrhization in tomato roots. Also, there were no variations in the mycorrhization intensity or the number of arbuscules inside colonized areas, indicating that the initiation and propagation of TYLCSV throughout the entire plant may not have a substantial impact on the intra-radical development of *F. mosseae*. According to Khoshkhatti et al. (2020), there was no noticeable distinction between healthy and TBSV/ToMV-infected tomato plants in terms of *R. irregularis* root colonization. Additionally, both healthy and PVY-infected potato plants showed the same F% level (Sipahioglu et al. 2009). This finding is in line with the up-regulation of five specified plant genes that were previously identified as mycorrhiza-responsive and selectively expressed in arbuscular-containing cells in both healthy and TYLCSV-infected cells (Fiorilli et al. 2009; Maffei et al. 2014).

However, Nemec and Myhre (1984) noted that, in contrast to diseased plants, the number of fungal spores and the proportion of mycorrhization were typically higher in healthy plants. The observation of Rùa et al. (2013) that *Barley yellow dwarf virus* (BYDV) and *Cereal yellow dwarf virus* (CYDV) infection increased F% of roots only under increasing CO_2_ concentration suggests that AM fungus and the virus collaborated to boost one another’s success. According to a study by Miozzi et al. (2020), when compared to healthy plants, AM fungal colonization in virus-infected plants showed a small increase in terms of mycorrhization intensity and the proportion of arbuscules throughout the entire root system. Contrary to these results, no statistically significant changes were seen in the frequency of mycorrhization or the quantity of arbuscules in the colonized segments of cucumber roots (Table 4). These findings suggest that the development of viral infection does not significantly affect AM fungal colonization.

### Viral detection by TEM and ELISA evaluation

**Fig. 5:**
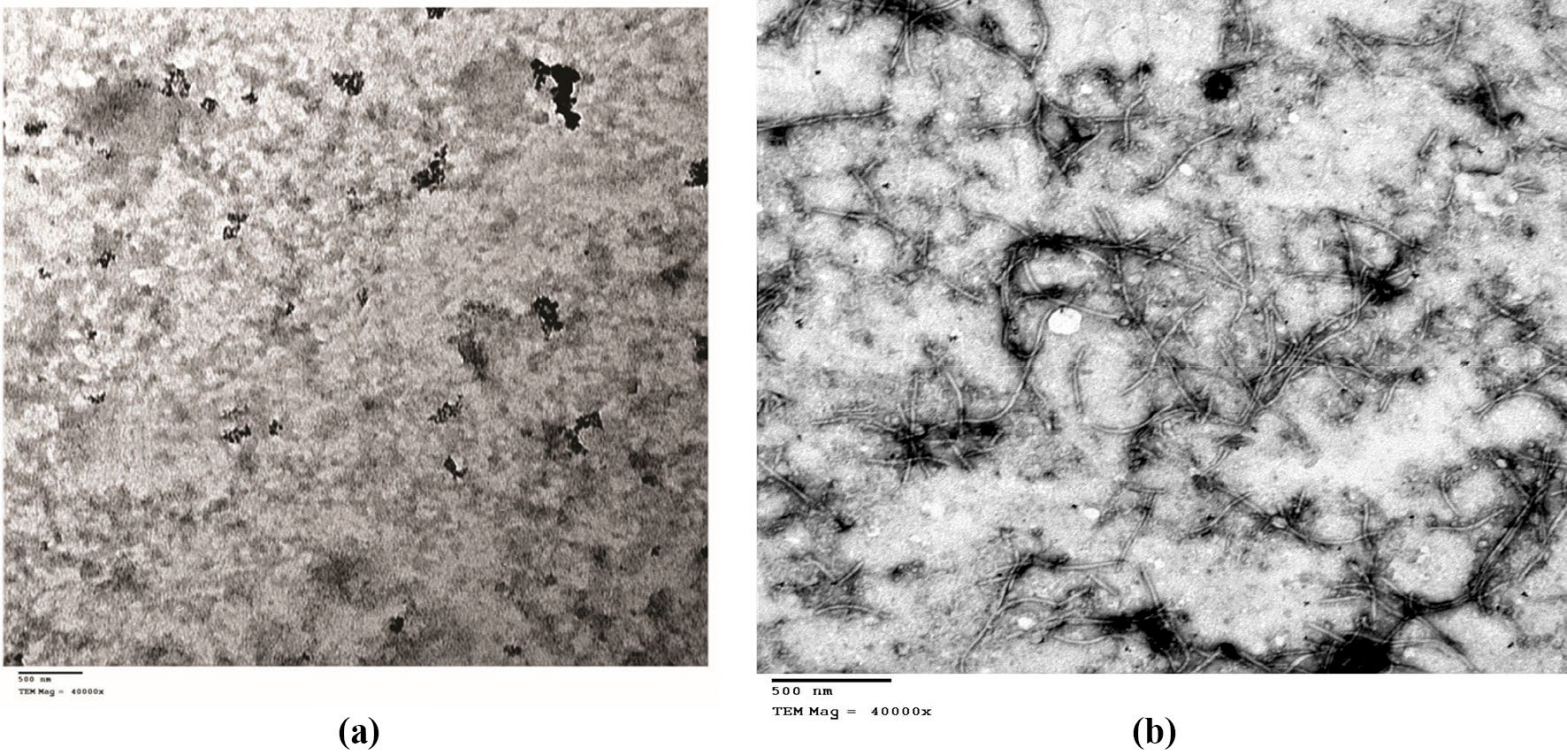
TEM images of the sap of control (**a**) and infected (**b**) cucumber leaves. Particles of ZYMV occur as filamentous flexible particles, which are 750 nm long and approximately 15 nm in width in the sap of infected cucumber leaves

Virus-infected leaves of cucumber plants showed severe symptoms, i.e. yellow mosaic, mottling and green blisters of leaves on the new leaves at 20 dpi (Fig. 2). The observations of purified healthy cucumber leaves using TEM examination revealed the absence of ZYMV viral particles (Fig. 5a). However, TEM examination of the partially purified infected cucumber leaves revealed the presence of long, filamentous, flexible viral particles of ZYMV with a width of 15 nm and a length of 750 nm which confirmed its relation to the *Potyviridae* family (Fig. 5b). As, Radwan et al. (2007) examined virus-infected leaves of pumpkin that exhibited severe symptoms on their leaves at 15 dpi under TEM and observed the presence of flexuous filamentous particles about 750 nm long and approximately 10 nm in width.

Based on ELISA results of ZYMV concentrations in cucumber leaves at 20 dpi, we observed that ZYMV-infected leaves gave the highest absorbance, which reflects the appearance of severe symptoms on cucumber leaves due to the high virus concentration (Table 2). However, in AM-inoculated plants, ZYMV concentrations were significantly lower than those of ZYMV-infected ones by 5.23-fold. Additionally, the mean absorbance values in HC plants, whether AM-inoculated or not, were almost zero, indicating symptomless. In healthy AM-colonized plants, the lowest virus concentration of 0.044 was found (Table 2). The ELISA results support the hypothesis that AM colonization played a protective function in reducing ZYMV infection and concentration in cucumber plants.

Elsharkawy et al. (2012) obtained similar results in cucumber when they applied dual inoculation of *Glomus mosseae* and *Fusarium equiseti* (GF18-3) to control CMV; the absorbance values of ELISA detection for *G. mosseae* and GF18-3-treated plants were significantly reduced as compared to *F. equiseti-*treated alone at 7 and 14 dpi. Most recently, in tomato plants, the efficient biocontrol activity of AM colonization against ToMV infection was validated by Mendoza-Soto et al. (2022), which led to a marked decline in the viral accumulation level. On the contrary, Fakhro et al. (2010) revealed that the utilization of *Piriformospora indica* as a biocontrol agent increased the accumulation of *Pepino mosaic virus* (PepMV). Miozzi et al. (2011) revealed a non-significant change in TSWV accumulation in tomato between non-colonized and *F. mosseae*-colonized plants at 14 dpi. In contrast, Khoshkhatti et al. (2020) reported a considerably greater ToMV concentration in AM-colonized plants as compared to non-AM ones at all dpi. This impact might be brought on by the elevated nucleic acid and protein output in these plants, which promotes viral multiplication and increases the spread of a virus across the entire plant (Dehne 1982), or it might be brought on by the increased phosphate uptake connected to plants that have been colonized by AM (Daft and Okusanya 1973).

### The effect of mycorrhization on MDA content under ZYMV infection

**Fig. 6:**
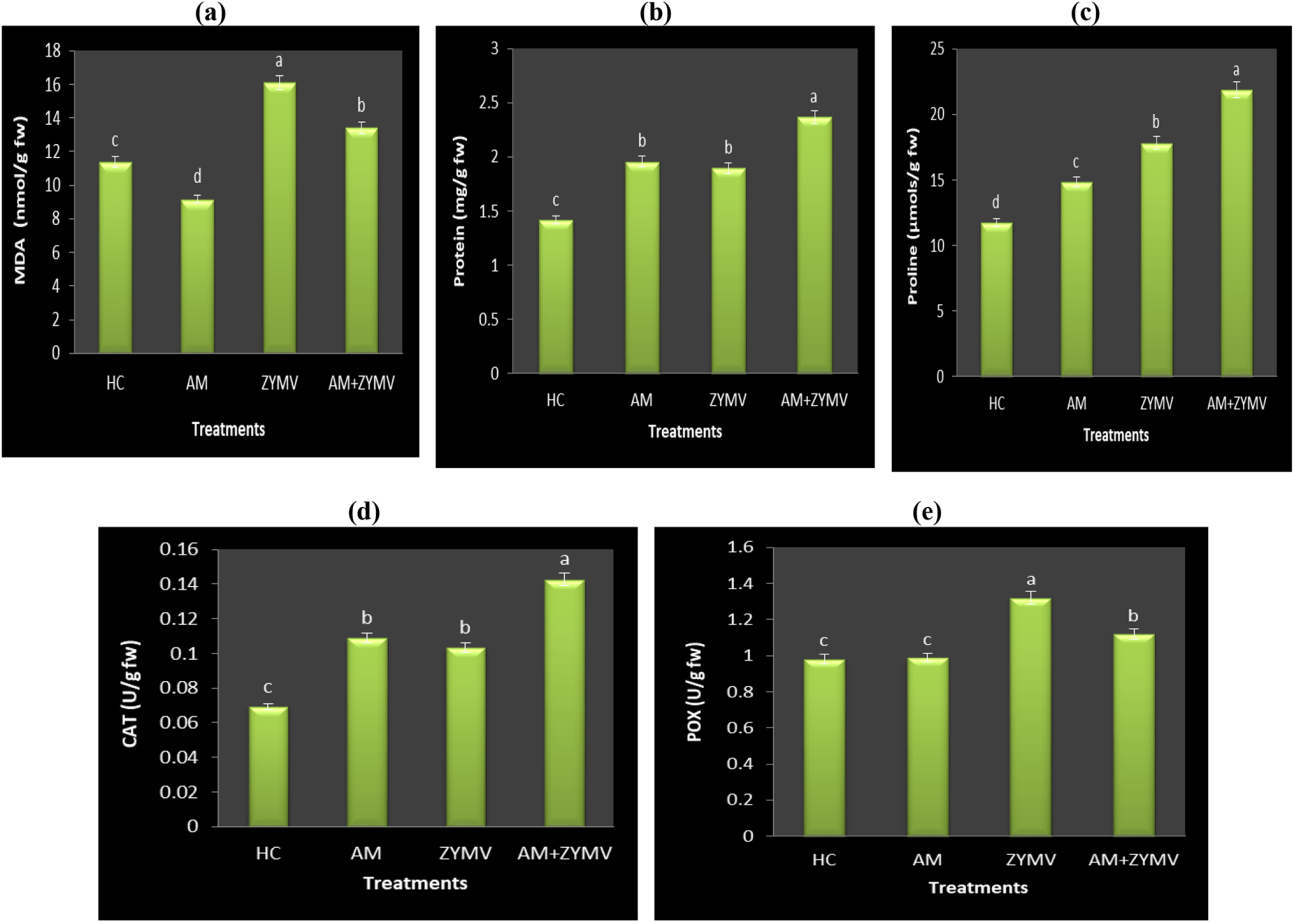
Effect of ZYMV infection and AM root colonization on the (**a**) MDA (nmol/g fw), (**b**) protein (mg/g fw), (**c**) proline (µmols/g fw) and antioxidant enzymes (CAT (**d**) and POX (**e**)) contents of cucumber leaves. HC = Healthy control, *AM* plants colonized with AM fungi, *ZYMV* ZYMV-infected plants and AM + ZYMV = AM and ZYMV-infected cucumber plants. *Values of each column followed by the same letter are not significantly different according to Duncan’s multiple range test (*p* ≤ 0.05), each value represents the mean of three replicates ± SE. a, b, c and d: symbols of significance letters

Defense reactions during plant-virus interactions are associated with the buildup of reactive oxygen species (ROS). It is significant to note that ROS have a dual function in inducing pathogen limitation and frequently localized death of host plant cells at the infection sites, as well as acting as a diffusible signal that triggers antioxidant and pathogenesis-related defense responses in surrounding plant cells (Hernández et al. 2016). So, a further experiment was conducted to better appreciate the oxidative damage through lipid peroxidation analysis as one of the biomarkers for oxidative stress measured by malondialdehyde (MDA) content (Abdelhameed et al. 2021b; Abdalla et al. 2022; Soliman et al. 2023). In the present work, ZYMV-infected cucumber plants accumulated more MDA (16.096 nmol/g fw) than healthy plants (11.387 nmol/g fw) (Fig. 6a). These results are in line with earlier studies, which found that MDA levels were greater in ZYMV- and *Mal de Rio Cuarto virus* (MRCV)-infected pumpkin and wheat plants, respectively (Radwan et al. 2007; Di Feo et al. 2010). Siddique et al. (2014) investigated how another viral disease, leaf curl disease caused by CLCuV, affected the leaves of two susceptible and resistant cotton genotypes, and found that after infection, the susceptible genotype leaves had higher MDA concentrations. Recently, Hamzah et al. (2021) revealed that *Bean yellow mosaic virus* (BYMV)-infected plants maximized the MDA content, showing that the MDA could be a great indicator of membrane disorder in plants exposed to pathogen infection (Loreto and Velikova 2001).

Conspicuously, with AM application, MDA content in cucumber leaves was significantly lower than that of non-colonized ones (Fig. 6a); where a lower MDA content was detected with AM application (13.419 nmol/g fw) in ZYMV-infected plants versus non-AM applied ones (16.096 nmol/g fw). The reason behind this might be the increased proliferation brought on by AM fungal colonization in AM-colonized plants as opposed to those non-colonized ones under viral stress, and this accelerated growth in AM plants could compensate for the damage caused by viruses (Hao et al. 2019), lowering MDA concentration.

### Effect of mycorrhization and ZYMV infection on protein and proline contents

Protein and proline contents of healthy, AM-inoculated and ZYMV-infected plant leaves are shown in Fig. 6b and c. The most noticeable result is the significant increase in both protein and proline contents in AM-inoculated and ZYMV-infected cucumber plants. AM fungal inoculation caused a significant increase in proteins (38.10 and 67.04%) and proline contents (26.30 and 86.12%) in healthy and ZYMV-infected plants as compared with the non-colonized healthy ones. Most extraordinarily, AM application seems to enhance proline and protein accumulation in AM-colonized as compared to non-colonized. These results are in accordance with the results obtained by Metwally and Abdelhameed (2018, 2019) under abiotic stresses.

Indeed, proline accumulation occurs in higher plants as a common response to abiotic and biotic stresses. For instance, pathogen infection (Soni et al. 2022), salinity (Metwally and Abdelhameed 2018), and high and low temperatures (Gosavi et al. 2014) may activate proline production. Our results are supported by Chatterjee and Ghosh (2008) and Gholi-Tolouie et al. (2018) findings of the increase in proline content in mesta and tomato plant leaves in response to MeYVMV and CMV. In Arabidopsis leaf tissues, the amount of free proline rises, inducing a hypersensitive reaction to pathogens (Fabro et al. 2004).

Similarly, a study by Abdelhameed et al. (2023) showed a significant increment in proline under the biotic stress of *Alternaria alternata*. Furthermore, in both healthy and ZYMV-infected plants, AM colonization can cause proline accumulation (Fig. 6b and c). As soon as plants are exposed to microbial pathogens, they release ROS that cause programmed cell death in the plant cells near the infection site, thereby fencing off the pathogen and stopping the disease process (Hamzah et al. 2021; Soliman et al. 2023). The amino acid proline functions as a powerful ROS scavenger, redox balancer, molecular chaperon, and protein structural stabilizer, which may inhibit the triggering of programmed cell death by ROS (Chen and Dickman 2005; Soni et al. 2022). Our results of protein accumulation under ZYMV infection were in line with those of Di Feo et al. (2010) due to the biotic stress of MRCV in wheat plants. Similarly, in response to ZYMV infection, Radwan et al. (2007) found a considerable rise in the amounts of soluble, insoluble, and total proteins in *C. pepo* leaves, and they also noted that the newly synthesized polypeptides that appeared in the ZYMV-infected sample are believed to be pathogenesis-related proteins.

### Effect of mycorrhization and ZYMV infection on defense antioxidant enzymes

Plant viruses, in contrast to bacteria and fungi, can reach the intracellular space of plant cells, where they are closely linked to the cytoplasm and cellular organelles, during viral pathogenesis and cause oxidative stress in the cell. Because antioxidant enzymes play a key role in scavenging ROS and lowering the oxidative stress on nucleic acids, proteins, and lipids, cells are equipped with these enzymes as a defense mechanism in plants to protect them from this stress (Metwally and Abdelhameed 2018; Nasrallah et al. 2020; Metwally and Soliman 2023). We therefore examined the antioxidant enzyme activity in both healthy and ZYMV-infected cucumber leaves (Fig. 6 d and e), focusing in particular on CAT and POX activity that catalyze H_2_O_2_ to H_2_O and O_2_. One-way ANOVA (*p* < 0.05) results highlighted that both AM and ZYMV treatments showed highly significant increases in POX and CAT activities, compared to HC. In ZYMV-infected leaves, their activities increase significantly (34.71 and 49.00%) compared to the HC; respectively (Fig. 6d and e). Our findings are consistent with those of Milavec et al. (2001) and Radwan et al. (2007), who found that potato and *C. pepo* leaves infected with Potato virus Y^NTN^ and ZYMV, respectively, had increased POX activity. Regarding CAT, Kobeasy et al. (2011) and Siddique et al. (2014) demonstrated an increase in its activity in response to *Peanut mottle* and CLCuV in *Arachis hypogaea* and cotton; respectively. A virus infection accompanied by an increase in cell metabolism increases the formation of ROS, which progressively destroys the cell compartments (Gholi-Tolouie et al. 2018). There are contradictory reports regarding a significant reduction in CAT activity in *Papaya Leaf Curl Virus* (PaLCuV) in Papaya-infected leaves (Soni et al. 2022) and *Phaseolus vulgaris* diseased with *White clover mosaic potexvirus* (WCIMV) (Clarke et al. 2002).

Moreover, the activity of both enzymes was higher in AM-inoculated cucumber leaves, either healthy or ZYMV-infected; with an increase of 105.96% for CAT and 14.04% for POX in ZYMV-infected leaves as compared to healthy, non-colonized ones (Fig. 6). According to this, AM symbiosis may reduce ROS damage by lowering MDA and by inducing more effective defense mechanisms to protect the host plant from the harmful effects of ZYMV. Because POX is frequently one of the enzymes that exhibits variations in activity under stress, Milavec et al.’s (2001) research demonstrated that POX participates in the plant's defense response to pathogen attack. It is closely linked to the enhanced capacity of systemically protected tissues to lignify as POX catalyzes the final polymerization phase of lignin formation, and this lignification process is thought to be a pathogen resistance mechanism during virus infection (Chittoor et al. 1999).

### Expression of the PR genes in cucumber plants

**Fig. 7:**
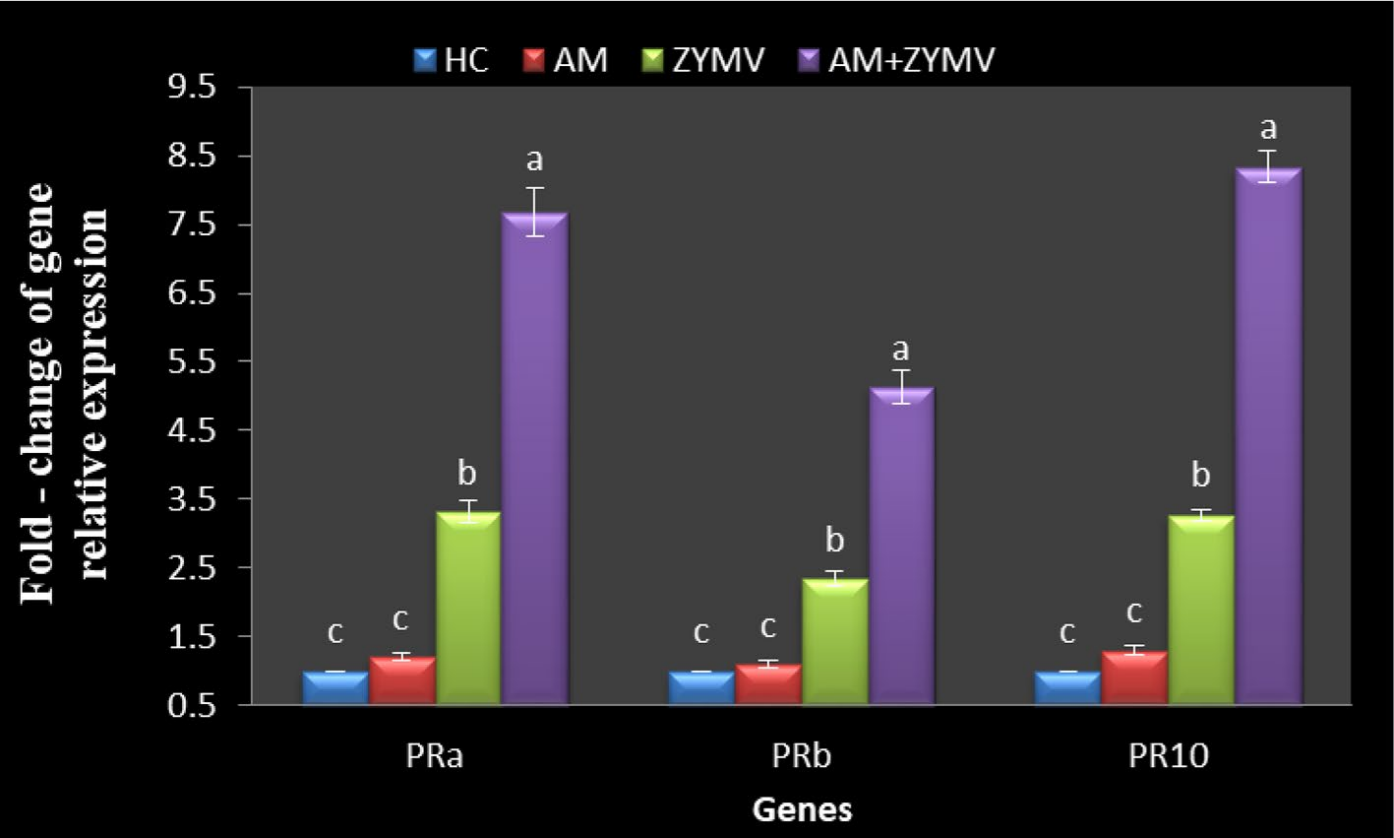
Effect of root colonization with AM and ZYMV infection on fold change of gene relative expression of cucumber leaves. *HC* Healthy control, *AM* plants colonized with AM fungi, *ZYMV* ZYMV-infected plants and AM + ZYMV = cucumber plants colonized with AM fungi and infected with ZYMV. *Values for each gene followed by the same letter are not significantly different according to Duncan’s multiple range test (*p* ≤ 0.05)

In this work, we discovered that the analyzed PR genes' expression levels were found following AM colonization of cucumber plants at 20 dpi of ZYMV. The ZYMV infection induced the relative expression of PRa and PR10 (by 3.3-fold), followed by PRb (2.3-fold increase). However, AM-colonized cucumber plants showed maximum expression of PRa and PR10 (by 8.3 and 7.7-fold increase), followed by PRb (5.1-fold increase) as compared to the HC ones (Fig. 7). According to Khoshkhatti et al. (2020), AM plants showed lower levels of TBSV virus accumulation and higher expression of PR genes, such as PR1, PR2, and PR3, at 20 dpi than TBSV-infected plants. Furthermore, Aseel et al. (2019) showed that AM colonization in the presence of ToMV increased transcriptional expressions of most of the studied genes, particularly PAL1 and HQT genes that regulate the first step in the main phenylpropanoid pathway, which is the start of the biosynthesis of many essential substances like flavonoids, coumarins, and lignans.

The mycorrhizal's beneficial effects on plant activities were clearly seen by reducing viral infection in the host photosynthetic process. In fact, genes associated with the photosystem II reaction center showed a widespread downregulation in cucumber plants infected with ZYMV, in contrast to plants colonized with AM, where a decrease in these parameters was scarcely perceptible. This matched the protective function of AM fungi that had already been noted in response to abiotic (Mathur et al. 2018) and biotic stressors (Aseel et al. 2019). In a comparable direction, Volpe et al. (2018) found that an elevation in salicylic acid (SA) during the early stages of AM colonization enhances siRNA-mediated antiviral silencing and primes SA-dependent defenses, the main defense mechanism against viruses.

In the current study with cucumber plants, the number of mycorrhiza-responsive genes was comparable to that previously reported for the tomato/*R. irregularis* interaction, where transcriptional changes related to the functional categories proteins, RNA, signaling, transport, biotic/abiotic stresses, hormone metabolism, as well as priming of systemic defense (Cervantes-Gámez et al. 2016). The AM can have a systemic impact on the plant's aerial portion even though it is only physically present in the root system (Cervantes-Gámez et al. 2016).

It was discovered that the PR proteins in the parts of the plant that were not infected prevented new viral infections and promoted resistance to viral replication and spread (Musidlak et al. 2017). By activating the production of PR-a, PR-b, and PR-10 proteins, which have ribonuclease and RNase activity in addition to their critical roles in stressors and antimicrobial action, the defense mechanisms in *Nicotiana benthamiana* leaves against beet necrotic yellow vein virus were promoted (Wu et al. 2014). Phosphorylation of the PR-10 protein was demonstrated to elevate ribonuclease activity to split viral RNAs (Musidlak et al. 2017). A strong activation of PR protein genes (PR1 and PR2) in AM-colonized plants infected with *Phytophthora infestans *in vitro (Gallou et al. 2011). Therefore, this work revealed that AM inoculation serves as an elicitor for the production of SAR in cucumber plants against ZYMV infection.

## Conclusions

The results of the present study indicate that AM root colonization and ZYMV infection exhibited significant alterations in the morphological and biochemical parameters of cucumbers. ZYMV initiated disease symptoms and decreased growth parameters and pigment fractions while increasing MDA content. Besides, it demonstrated the AM ability for tolerance induction against ZYMV-infected plants by alleviating disease symptoms and attenuating disease severity and viral concentration. Collectively, we present an overview of AM fungi's potential as bio-protectant agents that could assist cucumber plants in fighting ZYMV and strengthen their defenses by inducing proline, POX, and CAT activity and upregulating PR gene expression, providing the utilization of mycorrhizae as an elicitor for SAR against plant virus infection.

### Supplementary Information

Below is the link to the electronic supplementary material.

Supplementary file1 (DOCX 9094 KB)

## Data Availability

The relevant datasets supporting the results of this article are included within the article. https://www.ncbi.nlm.nih.gov/nuccore/OR474254.

## Abbreviations

AM: Arbuscular mycorrhiza
CAT: Catalase
dpi: Days post virus inoculation
DI: Disease incidence
DS: DISEASE severity
DAS-ELISA: Double Antibody Sandwich-Enzyme-Linked Immunosorbent Assay
HC: Healthy control
MDA: Malondialdehyde
PR: Pathogenesis-related
POX: Peroxidase
ROS: Reactive oxygen species
SA: Salicylic acid
SAR: Systemic acquired resistance
TEM: Transmission electron microscope
ZYMV: *Zucchini yellow mosaic virus*

## Declarations

### Experimental research and field studies on plants

All relevant institutional, national and international guidelines and legislation were compiled or adhered to in the production of this study.
