# Supplementary material for: Attenuation of Zucchini mosaic virus disease in cucumber plants by mycorrhizal symbiosis
Source: Plant Cell Rep. 2024 Feb 5;43(2):54. doi: 10.1007/s00299-023-03138-y (PMC10844420; doi:10.1007/s00299-023-03138-y)
Supplement: Supplementary file 1 — Supplementary file1 (DOCX 9094 KB) [file 299_2023_3138_MOESM1_ESM.docx]

| **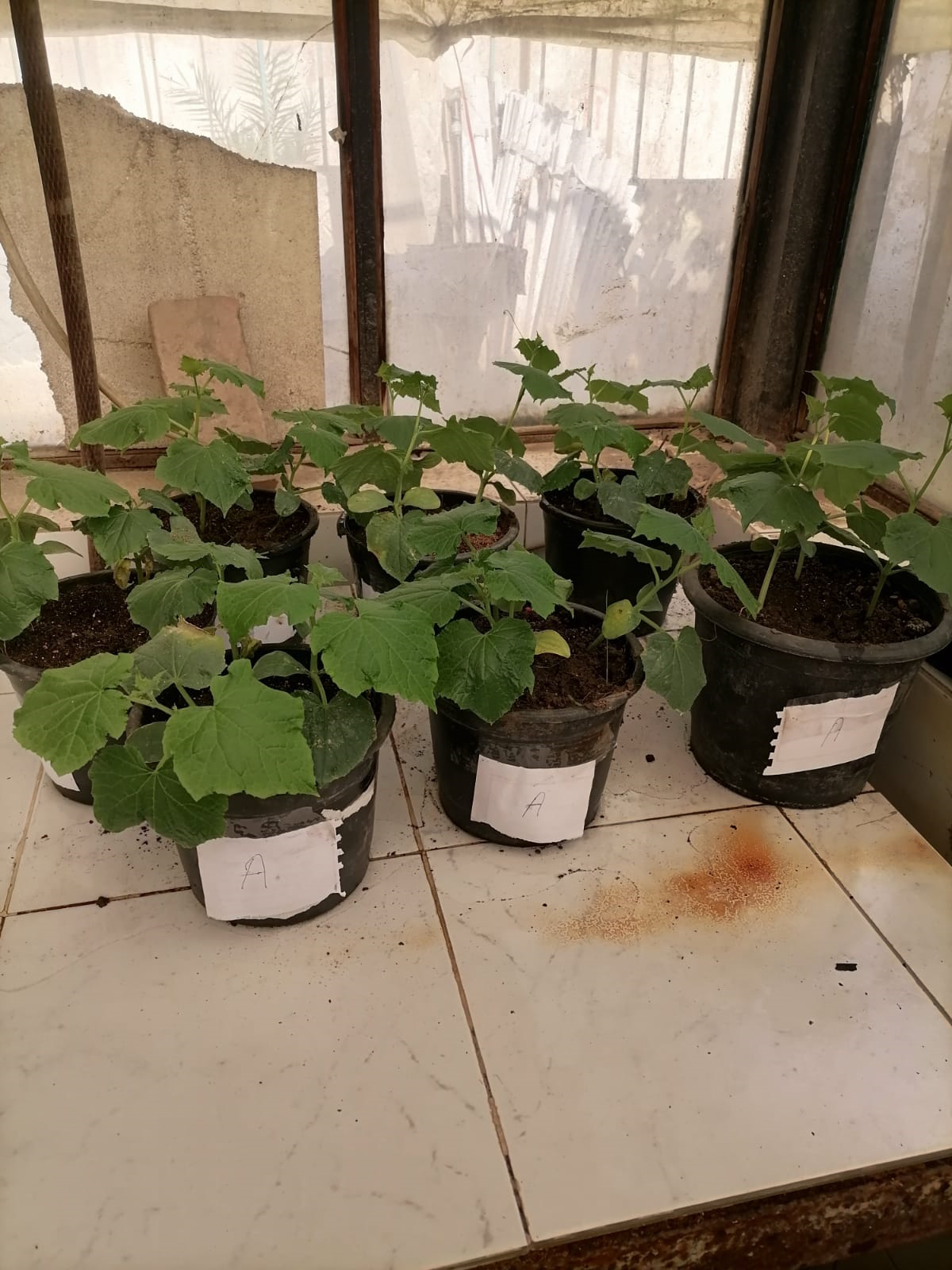**  **(a)** | | |
| --- | --- | --- |
| **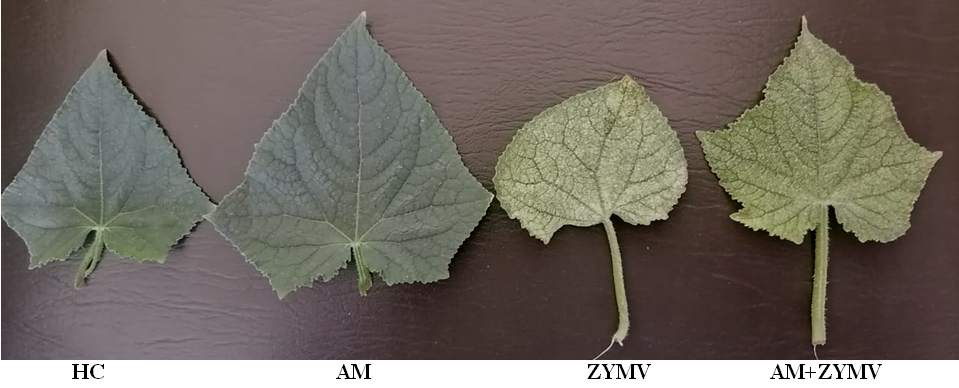**  **(b)** | **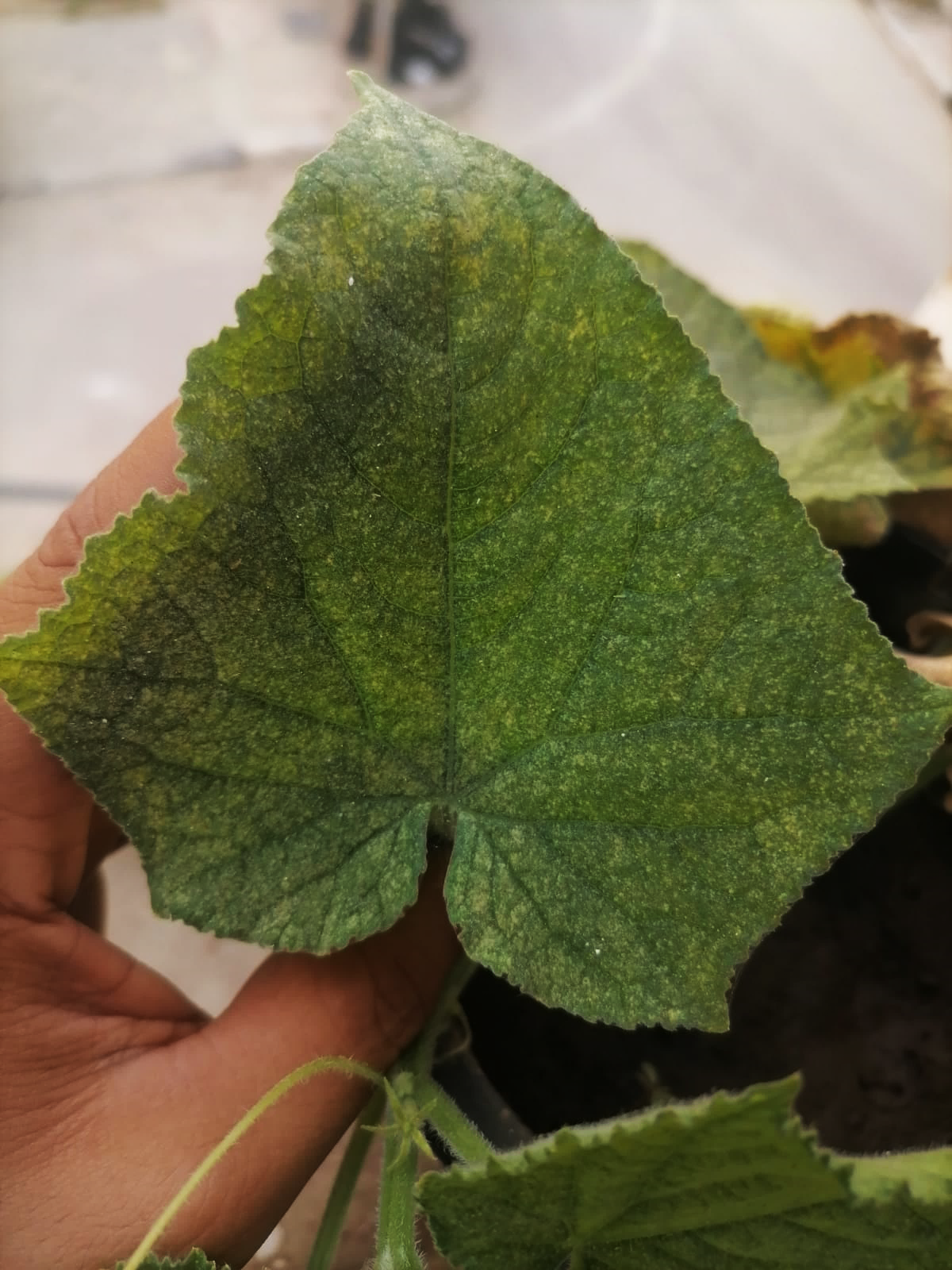**  **(c)** | **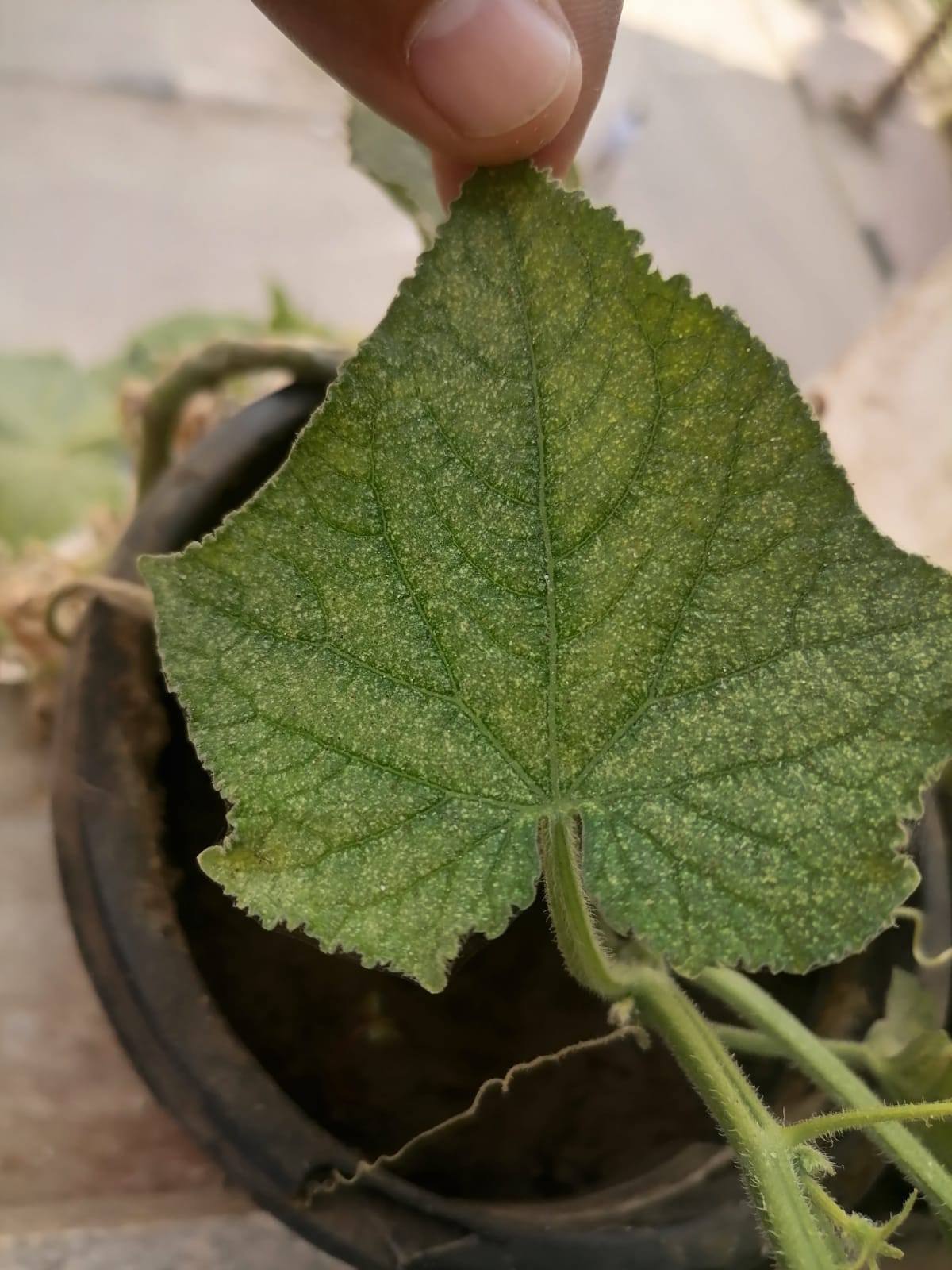**  **(d)** |

**Suppl. Figure 1.** (**a**) cucumber plants before ZYMV infection, (**b**) ZYMV symptoms in cucumber leaf after infection and (**c**) and (**d**) effect of AM on ZYMV symptoms.
